# The impact of trans fatty acids on ADHD in relation to the gut microbiome

**DOI:** 10.3389/fnut.2025.1641574

**Published:** 2025-11-19

**Authors:** Nianyang He, Jiayi Zhong, Songlin Deng, Jiyu Liang, Qingqing Li, Ke Chen

**Affiliations:** 1 Department of Child Health Care, Xindu Maternal and Child Health Care Hospital, Chengdu, China; 2 Department of Clinical Nutrition, Chengdu Women's & Children's Central Hospital, School of Medicine, University of Electronic Science and Technology of China, Chengdu, China; 3 Department of Rehabilitation, West China (Airport) Hospital, Sichuan University, Chengdu, China; 4 Department of Clinical Nutrition, West China Hospital, Sichuan University, Chengdu, China

**Keywords:** trans fatty acids, ADHD, intestinal flora, brain-gut axis, neurodevelopment

## Abstract

Attention deficit hyperactivity disorder (ADHD) is a neurodevelopmental disorder (NPDs) caused by the interaction of genetic and environmental factors. Trans-fatty acids (TFAs), mainly from chemically hydrogenated vegetable oils, are an environmental factor with increased exposure risk in modern diets. Recent studies suggest that TFAs may contribute to ADHD development through two pathways: inducing neurodevelopmental damage and indirectly affecting neural function by altering gut microbiota, though specific mechanisms remain unclear. This review focuses on two critical neurodevelopmental phases (perinatal period and adolescence) to explore the relationship between TFA exposure and ADHD, and to investigate the pathways through which TFAs affect ADHD by disrupting gut microbiota homeostasis. Although the association between TFA exposure during adolescence and ADHD is controversial, the harm of perinatal TFA exposure is undisputed. Shared neurodevelopmental damage mechanisms across both stages support reducing TFA intake during critical neurodevelopment. TFAs also impair neurodevelopment and brain function through the microbiota-gut-brain axis (MGBA) by disrupting gut microbiota homeostasis and activating neural, immune, and endocrine pathways. Thus, based on the harmful effects of TFAs during critical periods and the functional network by which TFAs contribute to ADHD pathogenesis through gut microbiota, this review supports dietary TFAs restriction as an ADHD prevention strategy.

## 1 Introduction

Attention deficit hyperactivity disorder (ADHD) is a prevalent neurodevelopmental disorder characterized by inattention, hyperactivity, and impulsivity, as well as abnormalities in emotion regulation that do not match the developmental stage (1). As reported by epidemiological surveys, ADHD affects 6.7%−7.8% of children and teenagers worldwide, and nearly half of the patients display symptoms that persist into adulthood, which is a huge public health problem (1). An essential factor in the occurrence and development of the disease is the interaction between genetic susceptibility and environmental exposure (2). As a vital component of the environment, the gut flora has attracted increasing attention due to its role in mediating neuroimmune-metabolic regulation through the gut-brain axis (GBA), a pathway of reciprocal interaction linking the gut microbiota to the central nervous system (3, 4). In current diets, the risk of exposure to industrial sources of trans fatty acid (TFA) resulting from artificially hydrogenated fats and oils has greatly increased (5). Epidemiological surveys have found that the total and industrially sourced TFA intake in preschool populations increased significantly, mainly due to increased consumption of ultra-processed foods, such as fast food, refined baked goods, and processed meat products (6). As shown in previous studies, TFAs as a typical component of ultra-processed foods, may affect neurodevelopment due to the following mechanisms (7). On the one hand, TFA exposure may result in learning and memory impairment in the brain through the following possible pathways: induction of oxidative stress, disruption of synaptic plasticity, enhanced neuroinflammatory responses, and vascular endothelial damage (7, 8). On the other hand, by affecting the composition of intestinal microbes, TFA may reduce the abundance of short-chain fatty acid (SCFA)-producing bacteria like *Bacteroidetes* and increase the proportion of pro-inflammatory bacteria such as *Proteobacteria* and *Desulfovibrionaceae*, further inducing intestinal barrier damage and systemic inflammation via the overproliferation of these pro-inflammatory bacteria (9, 10). Particularly, this gut dysbiosis may contribute to the pathogenesis of ADHD through multiple mechanisms. First, activation of the peripheral immune system leads to the releases inflammatory factors, including tumor necrosis factor-α (TNF-α) and IL-6, which in turn induce a central neuroinflammatory response by modulating the conduction of vagal nerve or blood-brain barrier (BBB) permeability; second, suppression of neuroprotective metabolites such as butyrate, results in dysfunction of the dopamine and serotonergic (5-hydroxytrytaminergic) systems; third, alterations in the bile acid metabolic profile impair the regulation of synaptic plasticity mediated by the nuclear receptor farnesol X receptor (FXR) signaling pathway (11–13).

Clinical findings suggest that children with ADHD have a much higher TFA intake and erythrocyte membrane TFA content than control subjects, with a positive correlation between the fatty acid content in the membrane and the severity of the disease (14, 15). Animal trials have shown that TFA exposure in Wistar rats results in an increase in oxidative stress markers in the prefrontal cortex and hippocampus, followed by mitochondrial dysfunction and heightened hyperactivity (16, 17). Earlier studies have demonstrated the harmful neurological effects of TFAs and their relation to the development of ADHD. Still, their susceptibility to exposure after prenatal life has not yet been reported and the exact mechanism by which TFAs impair the dynamic balance of the microbiota-gut-brain axis (MGBA) needs to be explored.

Therefore, this review will systematically discuss the impacts of TFA exposure through sensitive development windows and the interplay between gut microbiota disorders and ADHD pathogenesis, with a view to providing a theoretical basis for establishing ADHD prevention strategies involving dietary TFA restriction interventions.

## 2 An overview of ADHD

### 2.1 Key etiological influencing factors of ADHD

Research has reported that the male-to-female prevalence ratio of ADHD is 2–3:1 and that this ratio is most evident in childhood (18). Males have a higher content of androgens like testosterone, which may influence brain development and the behavior of children. This hormone may make it more difficult for boys to control their behavior and attention during the process of growth, increasing their risk of developing ADHD (19). These results above suggest that alterations in the endocrine system could play a major role in the emergence and progression of ADHD. Additionally, serum levels of the pro-inflammatory factor TNF-α were inversely associated with both symptom severity and gut microbiota diversity in ADHD patients (20). This correlation suggests that abnormal immune function and gut microbiota may contribute to pathological processes of ADHD by transmitting peripheral inflammatory signals to the central nervous system. However, epigenetic studies have demonstrated significant associations between DNA methylation modifications of LIME1 and SPTBN2 in children and attention deficit symptoms (21). In addition, the serotonin transporter gene (5HTTLPR) genotype and dopamine D4 receptor gene (DRD4) genotype were both significantly associated with susceptibility to ADHD symptoms, and the interplay between environment and genotypes is a key research direction for the non-genetic pathogenesis of ADHD (22, 23). As a whole, the above evidence suggests that the pathogenesis of ADHD involves the synergistic effect of genetic predisposition, epigenetic modulation, and environmental factors (toxins, diet) (2).

### 2.2 Limitations of current ADHD treatments and exploration of new strategies

Current first-line clinical medications, central stimulants such as methylphenidate and non-stimulants such as tomoxetine, can relieve the core symptoms of attention deficit in patients by inhibiting the dopamine transporter (24). However, approximately 10%−30% of patients will quit the medication due to the suboptimal response or side effects (25). Animal experiments have demonstrated that juvenile rats exhibit hypersensitivity to methylphenidate in the prefrontal cortex, and the treatment with MPH during the juvenile period has a long-term or lifelong effect on excitatory neuronal function in the prefrontal cortex (26). The current available evidence supports the safe use of short- to medium-term use of the drug. However, their potential to cause neurobehavioral problems and their long-term efficacy require further investigation (27). These limitations inspired the researchers to seek new therapeutic approaches, in which the regulatory strategy of the microbial-gut-brain axis (MGBA) has become a trending research topic (28). The gut microbiota, as the core part of MGBA, can regulate brain and cognitive development via neural, immune, and endocrine pathways (29). It has been found that gut microbiota dysbiosis and its metabolites cause local inflammation by disrupting the intestinal barrier and inducing elevated levels of inflammatory cytokines, immune cell infiltration, and abnormal vagal stimulation, ultimately leading to central inflammation and neurological dysfunction (30). This sequence of events further leads to brain functional deficits with neurodevelopmental disorders (NPDs) such as autism and ADHD (31). These findings have inspired the idea of dietary intervention to remodel gut microbiota and relieve ADHD symptoms.

## 3 ADHD influencing factors

### 3.1 How cognitive impairment affects ADHD development

The integrity of cognitive functioning is the most fundamental basis for normal brain function and social adaptability, and multidimensional cognitive impairments are present in patients with ADHD, such as executive dysfunction, attentional regulation deficit, and social cognitive deficit (32). This pattern of cognitive impairment not only significantly influences the clinical symptoms of the patients in the short term but also is closely related to their long-term prognosis. Executive dysfunction is the core pathophysiological defect of ADHD. It is characterized by reduced working memory capacity, impaired inhibitory control, and cognitive inflexibility (33). Studies on neurobiological mechanisms have found that the delayed prefrontal cortical development and abnormal activity of the basal ganglia-anterior cingulate gyrus circuit were the main causes of executive dysfunction in ADHD patients (34). A meta-analysis of relevant studies showed that approximately 30%−50% of ADHD patients still had executive function deficits in adulthood (35).

Research indicates that working memory capacity is positively correlated with academic performance in ADHD patients (36). Limited working memory capacity leads to persistent attentional regulation issues in these patients, causing frequent “cognitive breaks” that make it hard for them to consistently process instructions or task details, such as forgetting what teachers say or missing key information (37). In addition, impaired response inhibition is one of the core phenotypes of ADHD (38). In social settings, children with ADHD may face peer rejection due to impulsive behaviors, like interrupting others and not waiting their turn, leading to social conflicts and strained interpersonal relationships (39). Long-term social frustration may further impair interpersonal communication skills and trigger secondary emotional problems, such as low self-esteem and depression, thereby increasing disease burden (40).

Social cognitive deficits such as emotion recognition bias (e.g., misinterpreting the facial expressions of others) and impaired empathy (inability to perceive the intentions of others) are common in individuals with ADHD (39). This type of social cognitive impairment is associated with difficulties in interpersonal interactions, challenges in forming close bonds with peers, and an increased risk of social exclusion (41). If these deficits persist, they may result in a series of social problems in adulthood, including difficulties in daily life, poor occupational adaptation, and low economic status (42, 43). Cognitive impairment is now recognized as an important factor contributing to the poor long-term outcome of ADHD patients across neurobiological, behavioral, and social functioning dimensions (44).

Intervening in cognitive impairment is crucial for alleviating ADHD symptoms. Research indicates that enhancing executive function, especially working memory and inhibitory control, can reduce distraction and impulsivity. Existing intervention approaches are specifically designed to target this core objective. For instance, medications such as methylphenidate (a first-line medication for ADHD) modulate the dopamine system to improve cognitive resource allocation (45), while CBT provides compensatory strategies through targeted training to support this goal (46). In addition, childhood cognitive training and family behavioral management may reduce cognitive impairment in children and mitigate the long-term negative effects of ADHD (47). Furthermore, dietary intervention may also be effective in improving cognitive function. A meta-analysis of 10 trials involving 699 children found that Omega-3 fatty acid intervention exerted a moderate effect in alleviating ADHD symptoms compared with ADHD medications (48). In summary, individualized intervention strategies combining medication, psychological support, social support, and dietary strategies are of great importance to improve the cognitive function and long-term outcomes in ADHD.

### 3.2 The relationship between diet and ADHD

Recent research suggests that dietary components can affect the gut flora's shape and function via the MGBA, potentially contributing to ADHD development (49). Dietary patterns significantly affect the development of neurodevelopmental disorders by regulating gut flora and metabolism. In early childhood (0–3 years), gut flora is in the initial formation stage, and nutrient intake crucially influences its colonization. A study on children in Yaoundé showed that the balance of proteins, fats, and carbohydrates consumed during this period changes the gut microbiota's composition and functional traits (50).

Different dietary patterns have significantly different effects on ADHD risk. Preschool and school-aged children with ADHD often prefer a Western diet, high in saturated fats and low in fiber (51). A prospective study of 2,868 infants followed for 14 years found that higher Western dietary pattern scores were linked to elevated ADHD risk in individuals (52). Conversely, the Mediterranean diet, rich in whole grains, deep-sea fish, and polyphenols, with low saturated fat and abundant dietary fiber, may reduce ADHD prevalence and positively affect mental health in children and adolescents (53–55). The research into this phenomenon's mechanisms indicated that a Mediterranean diet rich in dietary fiber can promote the growth of SCFAs-producing bacteria, such as *Roseburia* and *Faecalibacterium* (56). SCFAs produced regulate neuroinflammation and the integrity of the blood-brain barrier by activating the G protein-coupled receptor (GPR43/41) signaling pathway (57, 58) and inhibiting histone deacetylase. Conversely, a diet heavy in fat and sugar leads to a decrease in intestinal flora α-diversity and disrupts the proportional balance between *Firmicutes* and *Bacteroidetes* (59), which may further lead to intestinal barrier impairment and inflammation (see Section 5.2 for detailed mechanisms) (60, 61).

Alongside dietary patterns, individual nutrients and food additives are also key factors in the development of ADHD. Various dietary fatty acids can influence cognitive performance in obese mice by altering intestinal inflammation and signaling pathways (62). Long-chain saturated fatty acids (LC-SFA), medium-chain saturated fatty acids (MC-SFA), n-6 polyunsaturated fatty acids (n-6 PUFA), monounsaturated fatty acids (MUFA), as well as trans-fatty acids (TFA) may impair cognition, while n-3 polyunsaturated fatty acids (n-3 PUFA) could offer protective benefits (62). Interestingly, an elevated maternal omega-6/omega-3 fatty acid ratio may raise the likelihood that a child would experience long-term ADHD symptoms (63). Furthermore, supplementing children with ADHD with omega-3 fatty acids resulted in a significant increase in EPA and DHA levels in their erythrocyte membranes. This change relieved the symptoms of inattention, hyperactivity, and oppositional behavior and improved working memory function (64, 65). Deficiencies in micronutrients, such as iron, magnesium, and vitamin D, may induce ADHD by interfering with dopaminergic signaling, neurodevelopment, and immunoregulatory processes (66–68).

Food additives are another potential risk factor for ADHD that should not be overlooked (69). A positive association between the consumption of snacks containing synthetic colors (lemon yellow and sunset yellow) and preservatives (sodium benzoate), and the appearance of symptoms in some children with ADHD has been demonstrated (70, 71). In addition, Benoit et al. found that artificial emulsifiers like carboxymethylcellulose and polysorbate-80, which are common in foods like cakes and ice creams, can alter gut microbiota, compromise intestinal barriers, promote lipopolysaccharide migration into the bloodstream, and trigger neuroinflammation in mice (72).

In summary, dietary factors such as dietary patterns, nutrient intake, and food additives can affect the brain via a variety of routes, including regulating intestinal flora, influencing short-chain fatty acid metabolism, altering intestinal permeability, and being mediated by inflammatory cytokines. These findings provide new insights into the pathogenesis of ADHD.

## 4 Effects of trans fatty acids on ADHD

### 4.1 Neurotoxicity of trans fatty acids

The brain is highly lipophilic, with 40%−60% of its dry weight being lipids—this high lipid content and lipophilicity allow TFAs to cross the blood-brain barrier, and thereby affect the nervous system through multiple mechanisms (73). TFAs have been suggested as contributors to neurological disorders like depression and Alzheimer's disease (74–76). A clinical trial found higher TFA levels in children with ADHD compared to healthy controls, indicating a potential link between TFA exposure and ADHD (15). Follow-up research has since demonstrated that the neurological effects of TFAs may be mediated by dopaminergic neurotransmission and that TFA-rich diets reduce dopamine levels and cause signaling abnormalities in the limbic system, ultimately impairing signaling along the dopamine reward pathway (77, 78). This signal transmission interruption is associated with motivational deficits and inattention in ADHD patients (79, 80). In addition, TFAs inhibit the synthesis of long-chain polyunsaturated fatty acids (LC-PUFAs) and disrupt lipid distribution in brain cell membranes (81). However, supplementation with LC-PUFAs may alleviate ADHD symptoms in children and adolescents (82).

In terms of oxidative stress, high TFA intake increases the activity of NADPH oxidase and the expression of inflammatory cytokines, thereby elevating oxidative stress (83). An animal study demonstrated that a high-TFA diet increases protein carbonyl (PC) levels in mouse brains, exacerbates neuronal oxidative damage and may further impair memory function (84).

Regarding gut-brain interactions, TFAs induce intestinal dysbiosis, increasing the proportional abundance of pathogenic bacteria *Proteobacteria* and *Desulfovibrionaceae*, while decreasing the proportional abundance of beneficial bacteria *Bacteroidetes* and *Lachnospiraceae* (9). This microbial shift may cause alterations in the metabolic activities of intestinal microbiota, resulting in a decrease in the overall quantity of short-chain fatty acids. As metabolites produced by the gut microbiota during the fermentation of dietary fiber, short-chain fatty acids (SCFAs) can regulate nervous system function through binding to cell surface receptors. For example, the SCFA propionic acid modulates neuroinflammation and maintains the blood–brain barrier integrity by activating free fatty acid receptor 2 (GPR43) (85). Thus, TFAs can also indirectly affect nervous system function by reshaping the composition and metabolic activity of gut microbiota.

### 4.2 Developmental window sensitivity

During different developmental stages, including the perinatal period (28 weeks of gestation to 1 week postpartum), infancy, childhood, and adolescence, the body's sensitivity to exposure to TFAs varies significantly. The perinatal period is a sensitive time for central nervous system ontogeny. A mother's dietary pattern may influence the structural brain development and behavioral outcomes of her children (86). Animal studies showed that excessive TFA intake in mother rats enabled TFAs to cross the placental barrier into offspring, altering the fatty acid composition and oxidative status in the brain of offspring and reducing the expression of TrkB, and this reduction could directly lead to the failure of the neuroprotective function of brain-derived neurotrophic factor (BDNF) (87, 88). The abnormal changes in these intracerebral physiological indicators may disrupt the developmental neurological trajectory, affect brain function, and lead to memory disorders (88). Other research suggests that high maternal consumption of TFAs, palm oil, or esterified fats during pregnancy and lactation may trigger brain inflammation in offspring by disrupting neuroactive compound balance (89). This can harm brain functions related to cognition and mood (90).

Perinatal TFA exposure affects offspring neurodevelopment directly and indirectly by disrupting their gut microbiota. A high-fat maternal diet reshapes maternal tract flora, disrupting the embryonic brain's glutamate-glutamine cycle, thereby increasing the expression of several glutamate-related genes (86). This disturbance may not only persist into adolescence but also trigger gender-dependent hyperactivity and anxiety-like behaviors. Interestingly, it has been found that high TFA exposure in pregnancy induces an inflammatory response in the colon of the offspring. This might be connected to a change in the offspring's gut microbiota. Maternal dietary supplementation with Jussara is effective in mitigating this colonic inflammatory response in the offspring and improving their gut health (91).

The TFA content of breast milk, a critical source of nutrition during early infancy, is affected by the mother's dietary composition (92). TFAs in breast milk may be transferred to infants and inhibit the synthesis of LC-PUFAs in infants (81). LC-PUFAs are critical to synaptic development and neuronal myelination. Metabolic disorders of LC-PUFAs may hinder the development of infants' neurons and increase the risk of neurodevelopmental disorders, such as ADHD in these infants (93).

During childhood and adolescence, the effects of TFAs differ from those in the perinatal stage. An animal study found that high TFA intake impairs spatial memory in young rats (94). In children, high consumption of saturated and trans fats is linked to cognitive decline related to the hippocampus, while omega-3 exert a positive effect (95). Prolonged TFA exposure in young animals leads to reduced motor activity and exploratory behavior, as well as increased fear and anxiety-like behaviors (96). This indicates that prolonged exposure to TFA during childhood may aggravate neuromotor dysfunction and neuropsychiatric behavioral problems. In adolescents, TFAs may modulate the brain's oxidative status by inducing lipid peroxidation and lowering antioxidant enzyme activity, ultimately leading to anxiety behaviors (97). In addition, TFA intake could raise the probability of metabolic syndrome, promote systemic inflammatory responses, and enhance blood-brain barrier permeability, which may be detrimental to cognitive development in a dual manner (98–100). Although many studies have confirmed the negative neurological effects of TFAs at different developmental stages, the link between maternal TFA exposure and ADHD in children and adolescents remains debated. Maternal TFA exposure during the perinatal period is associated with smaller fetal head circumference in late pregnancy, though its effect on fetal head size in mid-pregnancy and whole-brain volume at age 10 remains unclear (101). Therefore, based on the negative impacts of TFAs on neurodevelopment during critical developmental windows and their association with ADHD-related phenotypes, reducing TFA intake from the perinatal period to adolescence may serve as one of the strategies to prevent ADHD onset. However, population-based evidence directly linking TFA exposure at different developmental stages to ADHD onset is relatively limited, with most conclusions from animal experiments. In the future, the effectiveness of this strategy requires further verification through more population-based studies and the multifactorial etiological characteristics of ADHD should also be considered.

## 5 Mechanisms of trans fatty acids effects on ADHD

Even though some countries have restricted TFA use in industrial foods, TFA consumption is expected to increase with the growing prevalence of home baking and the expanding variety of processed food items. After ingestion, TFAs enter the human body and are digested and absorbed in the intestines. Emerging evidence suggests they may affect the occurrence and development of ADHD through potential mechanisms such as influencing the function and development of the nervous system and disrupting the gut-brain axis. Their potential mechanisms are summarized below.

### 5.1 How trans fatty acids indirectly trigger ADHD through brain-mediated factors

#### 5.1.1 Trans fatty acids lead to impaired metabolism of essential fatty acids

A birth cohort study in Spain found that a higher omega-6:omega-3 ratio in prenatal cord plasma is linked to subclinical ADHD symptoms in children (63). Therefore, the balance between omega-3 and omega-6 fatty acids is of great importance in the development of neurological function. Maternal diet, breast milk, and the concentration of TFA in infant plasma phospholipids are all positively correlated, while TFA in breast milk is negatively correlated with the levels of essential fatty acids 18:2n-6 (linoleic acid, LA) and 18:3n-3 (α-linolenic acid, ALA) (102). That is to say, when the content of TFA in breast milk increases while the n-3 PUFA content decreases, this may be attributed to TFA disturbing the balance of essential fatty acids in the body. Dietary n-3 PUFA supplementation reduces the accumulation of TFA in the brain and enhances the accumulation of DHA (103). DHA, an omega-3 fatty acid, is vital for neuronal and retinal membrane integrity, neural signaling, and brain energy metabolism. Its deficiency leads to alterations in learning ability, stress response, and behavior (104). Additionally, Lara et al. discovered that omega-3 PUFAs supplementation alters gut microbiota by increasing *Bacteroidetes* and butyrate-producing *Lachnospiraceae*, while decreasing Faecalibacterium, leading to more anti-inflammatory compounds like short-chain fatty acids (105). This suggests that omega-3 PUFAs may act on the gut-brain axis by altering the composition of the gut microbiota.

#### 5.1.2 Effect of trans fatty acids on fat-soluble vitamins

TFA exposure jeopardizes neurological health by disturbing the metabolic homeostasis of vitamin E and vitamin D through oxidative stress and organ damage pathways. High TFAs intake increases oxidative stress by promoting NADPH oxidase activity and inflammatory cytokine expression (83). As a key antioxidant, vitamin E supplementation reduces oxidative stress in humans (106), implying that TFA-induced oxidative stress may accelerate vitamin E depletion and that vitamin E supplementation could mitigate such TFA-mediated oxidative stress. In addition, TFAs lead to hepatic fat accumulation and non-alcoholic steatohepatitis-like damage, thereby impairing liver function (107). As the liver is the main storage organ for vitamin E, hepatic steatosis further reduces vitamin E storage, metabolism, and conversion efficiency (108). Chronic vitamin E deficiency not only aggravates oxidative damage in the brain and increases the risk of cognitive impairment, but also disrupts gut flora and imbalances the ratio of *Firmicutes* to *Bacteroidetes* (109, 110). Research has shown that abnormalities in the gut microbiome are key factors in the onset of neurological disorders, including ADHD (111).

Research indicates that low vitamin D levels in obese children are linked to increased oxidative damage and inflammation (112), suggesting a harmful interaction between inflammation and vitamin D deficiency. This deficiency can downregulate the transcription of the antimicrobial peptides cathelicidin and defensin beta 4, which in turn leads to reduced intestinal flora diversity, impaired intestinal function, and increased pathogen growth, creating an “inflammation-intestinal flora dysbiosis vicious cycle” (113). Clinical research shows that children with ADHD have lower vitamin D levels, and vitamin D supplementation markedly attenuates inattentive and impulsive symptoms (114). Additionally, vitamin D deficiency during early pregnancy may raise the risk of ADHD in offspring (115), highlighting its protective role in neurodevelopment.

In summary, reducing TFA intake (e.g., limiting fried foods and processed snacks) while supplementing with vitamin E (through dietary nuts and leafy greens) and vitamin D (through deep-sea fish and sunlight exposure) may reduce the risk of neurodevelopmental abnormalities through multiple pathways, including antioxidant, anti-inflammatory, and microbiota regulation.

#### 5.1.3 Effect of trans fatty acids on essential elements

Iron, magnesium, and zinc are essential elements for nervous system health. Studies show children with ADHD have lower levels of these elements compared to healthy peers, suggesting a link between deficiencies in these elements and ADHD (116–118). Zinc is vital for neurotransmitter synthesis and regulation (119), while magnesium exerts neuroprotective benefits by influencing ion channels and neurotransmitter activity (120). Iron is widely present in the brain and involved in processes such as neuronal development, myelin formation, and neurotransmitter synthesis (121). Chen et al. reported that iron content was decreased in dopamine transmission-related brain regions, including the bilateral striatum, anterior cingulate, cortex, and olfactory gyrus, in ADHD children, while iron content in the left anterior cingulate was positively correlated with ADHD symptom severity (122). In addition, altered brain iron levels in children are associated with impaired sustained attention, thus further corroborating the crucial role of iron in ADHD pathophysiology (123).

TFAs may hinder the absorption of essential minerals through various mechanisms. They can alter the expression of zinc-binding proteins at the cellular level, thereby affecting intracellular zinc levels (124). Additionally, TFAs alter gut microbiota composition, increasing harmful bacteria such as Proteobacteria and decreasing beneficial ones like Bacteroidetes (9), which disrupts intestinal balance and mineral absorption (125). Consequently, TFAs may reduce mineral absorption efficiency, thus affecting their normal utilization in the body and potentially worsening ADHD symptoms.

### 5.2 How trans fatty acids indirectly induce ADHD through gut microbiota–mediated mechanisms

Gut microbiota interacts with the central nervous system via the microbial-gut-brain axis, thereby regulating neurodevelopment and functional maturation (126). It exerts its effects through the immune, neural, and endocrine pathways, and there is cross-talk between these three pathways. It is important to note that TFAs may alter the composition and structure of gut microbiota, which in turn acts on the brain via the gut-brain axis and affects patients with ADHD.

#### 5.2.1 Activation of neural pathways by trans fatty acids via intestinal flora MGBA

**Figure 1:**
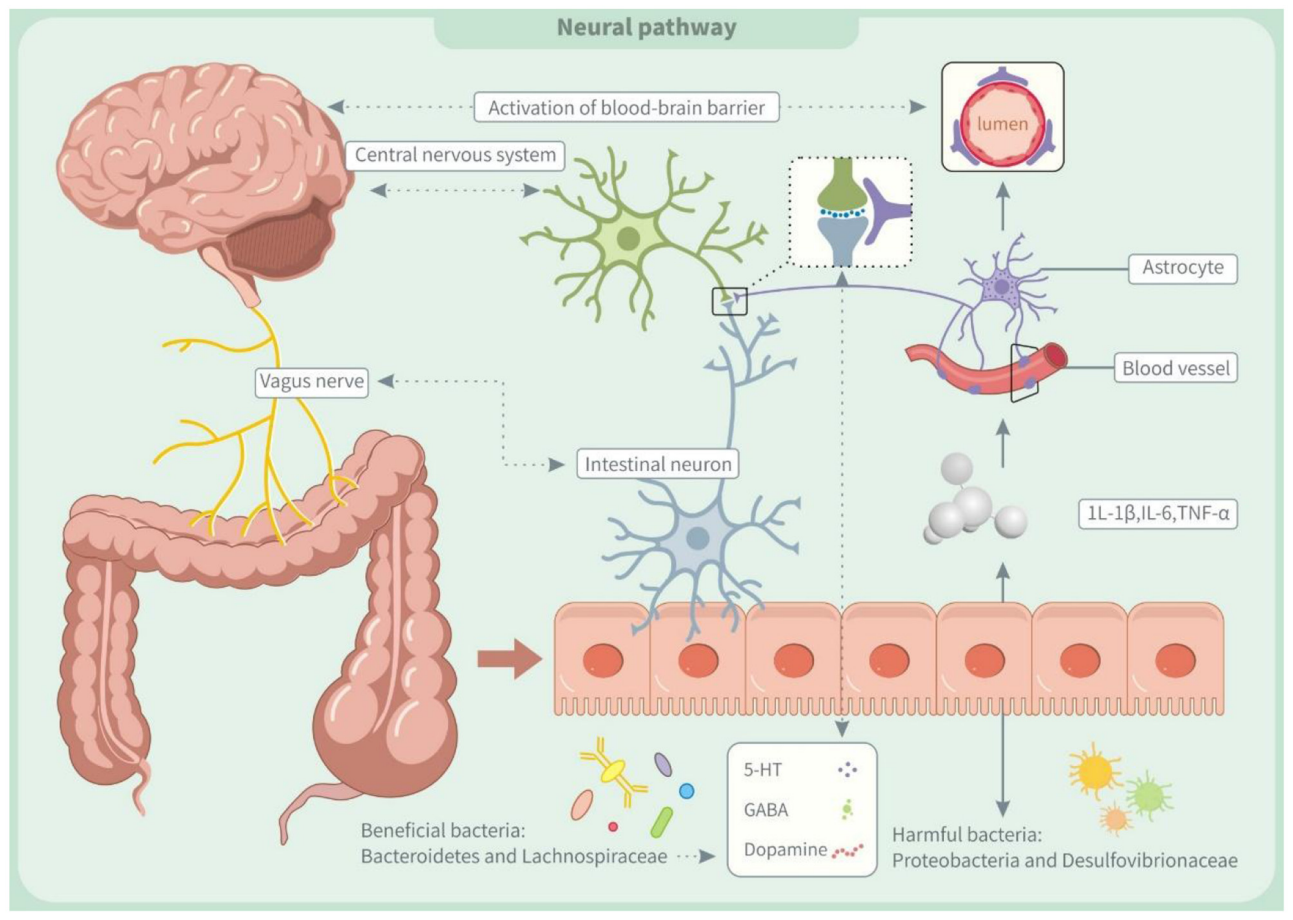
Trans fatty acids affect neural pathway through the microbiota-gut-brain axis (MGBA). IL-1β, interleukin-1β; IL-6, interleukin 6; TNF-α, tumor necrosis factor α; 5-HT, 5-Hydroxytryptamine; GABA, γ-aminobutyric acid.

Our findings in Figure 1 suggest that TFA exposure may regulate the neuroinflammatory process by remodeling gut microbiota structure and disrupting its metabolic function. Research has reported that TFAs can induce the proliferation of harmful bacteria like *Proteobacteria* and *Desulfovibrionaceae*, inhibit the proliferation of beneficial bacteria like *Bacteroidetes* and *Lachnospiraceae*, and cause intestinal microecological imbalance and intestinal inflammation (9).

Additionally, TFAs impede the conversion of intestinal acetate to butyrate, a mechanism that not only impairs intestinal barrier function but also exacerbates the body's inflammatory response by increasing bacterial translocation (127). The increase of inflammatory mediators like interleukin-1β (IL-1β), interleukin-6 (IL-6), and tumor necrosis factor-α (TNF-α) might negatively impact the central nervous system (128). On the one hand, inflammatory factors can disrupt the blood-brain barrier (BBB), allowing harmful substances to penetrate the brain and disrupt nerve cell function (129). On the other hand, it damages the autonomic nerves through the organ-brain axis, leading to neuroinflammation (130). Recent studies suggest that neuroinflammation and gut microbiota imbalances may contribute to ADHD by disrupting neurotransmitter development, affecting the synthesis of dopamine, norepinephrine, and BDNF (131, 132). These neurotransmitter abnormalities are closely linked to ADHD symptoms and their deficiency may worsen the condition (2).

#### 5.2.2 Trans fatty acids activate immune pathways via gut microbiota MGBA

Trans fatty acids contribute to the pathogenesis of neurodevelopmental diseases like ADHD by remodeling gut microbiota structure and activating immunological and neuroinflammatory pathways (2). In animal models, dysbiosis of intestinal flora induces intestinal oxidative stress, which increases HNE (4-hydroxynonenal) adducts while decreasing tight junction protein ZO-2. These changes stimulate the NF-κB pathway, leading to the generation of systemic autoantibodies and an immunological response, which suggests an interaction between gut microbiota and the immune system (133, 134). Notably, maintaining early gut microbiota balance is crucial for proper immune development. Dysbiosis in the newborn gut disrupts early gut microbiota balance, affecting the development of gut-associated lymphoid tissues and causing a systemic immune response in adulthood (135). This process may occur because flora disturbances weaken the mucosal barrier, allowing bacteria and metabolites to enter the body and disrupt the immune system (136, 137).

**Figure 2:**
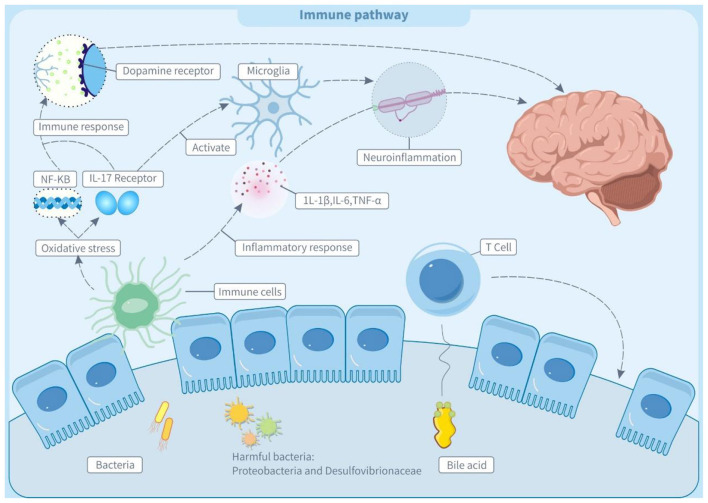
Trans fatty acids affect immune pathway through the microbiota-gut-brain axis (MGBA). IL-1β, interleukin-1β; IL-6, interleukin 6; IL-17 receptor, Interleukin-17 receptor; TNF-α, tumor necrosis factor α; 5-HT, 5-hydroxytryptamine; GABA, γ-aminobutyric acid; NF-kB, nuclear factor - kappa B; T cell, T lymphocyte.

As shown in Figure 2, all the above immune abnormalities may affect the nervous system through two pathways. On the one hand, a possible autoimmune reaction may lead to the production of autoantibodies against neural proteins (such as dopamine receptors), which disrupts the normal transmission of neurotransmitters and causes abnormalities in the nervous system (138). On the other hand, gut microbiota dysbiosis may interfere with the differentiation of Th17 cells, increasing IL-17A (interleukin-17A) levels. It has been reported that this cytokine can activate microglia and further aggravate neuroinflammation, which is an important risk factor for the occurrence and development of ADHD (139, 140).

The bile acid metabolism axis plays a key regulatory role in the molecular mechanisms of microbiota-immunity interactions. Secondary bile acid isoDCA (iso-deoxycholic acid) maintains intestinal immune homeostasis by antagonizing the farnesol X receptor (FXR) signaling pathway, simultaneously inhibiting the pro-inflammatory function of dendritic cells, and inducing the differentiation of regulatory T cells (pTreg) (141). TFAs may disrupt this negative feedback mechanism by reducing the abundance of isoDCA-producing bacteria such as *Bacteroides* (142).

#### 5.2.3 Activation of endocrine pathways by trans fatty acids via gut microbiota MGBA

**Figure 3:**
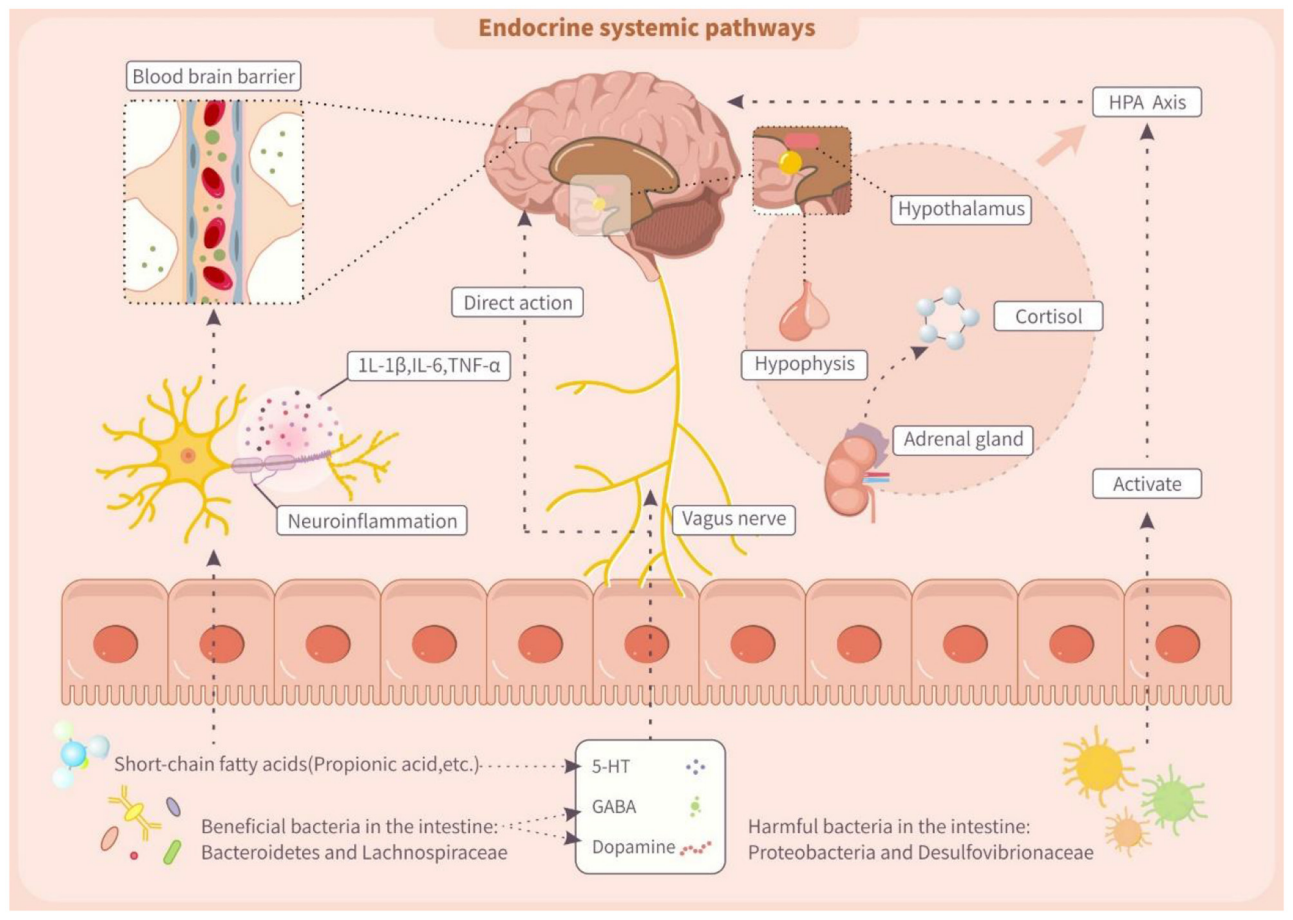
Trans fatty acids affect endocrine systemic pathway through the microbiota-gut-brain axis (MGBA). IL-1β, interleukin-1β; IL-6, interleukin 6; TNF-α, tumor necrosis factor α; 5-HT, 5-hydroxytryptamine; GABA, γ-aminobutyric acid.

Figure 3 (MGBA) suggests that TFAs can alter gut microbiota, thereby impacting neuroendocrine signaling in the hypothalamic-pituitary-adrenal (HPA) axis and central nervous system. Gut microbiota, a crucial participant in MGBA signaling, directly regulates the metabolism of neurotransmitters, including dopamine, 5-HT, GABA, and glutamate (143). Dopamine and 5-hydroxytryptamine are crucial for the executive function of the prefrontal cortex. Notably, dopamine affects cognitive modulation, reward processing, and motivation. Intestinal flora like *Bacteroides* and *Lactobacillus* contain dopamine synthesizing and catabolic enzymes, and can modulate their bioavailability (144). Furthermore, the intestinal flora regulates 5-HT synthesis and metabolism, which in turn influences intestinal motility and the emotional and cognitive functions of the central nervous system (145). This process may be modulated by its metabolites, SCFAs, that have effects on colonic enterochromaffin cells and modulate colonic 5-HT synthesis (146). Additionally, *Bacteroides* and *Lactobacillus* produce GABA, which influences intestinal integrity and vagal signaling, and when intestinal flora dysbiosis causes abnormalities in GABA synthesis or function, the imbalance of this pathway can potentially lead to neurological disorders (147, 148). Notably, TFAs can alter gut microbiota composition, potentially impairing SCFA production and disrupting HPA axis regulation (149). In animal studies, for example, gut dysbiosis was linked to reduced hypothalamic glucocorticoid levels and increased CRH synthesis, leading to HPA axis hyperactivation and elevated cortisol (150). This finding suggested gut microbes influence HPA axis activity. Clinically, children with ADHD exhibited lower HPA axis activity and cortisol levels, possibly due to gut microbiota dysregulation (151). Therefore, TFAs might activate endocrine pathways and affect HPA axis function by modifying gut microbiota.

Ultimately, TFAs may contribute to ADHD by disrupting gut microbiota balance and activating neural, immune, and endocrine pathways, suggesting they are potential environmental risk factors for ADHD. Further clinical studies are necessary to confirm causality, assess intervention effects, explore dose-response relationships, determine safe exposure levels, and develop prevention and treatment strategies for ADHD.

## 6 Conclusion and outlook

MGBA is a complicated communication network linking the gut microbiome and central nervous system, and its role in neuropsychiatric disorders like ADHD has gradually attracted attention. Focusing on exposure to industrially sourced TFAs, this review clarifies TFA impacts on ADHD across key growth stages and explores the potential mechanism by which TFAs affect ADHD through the modulation of gut microbiota by MGBA. This article also offers new viewpoints on ADHD etiology and highlights the importance of diet and environment for the neurodevelopmental health of children.

The impact of TFA exposure in the developmental window presented in this review has been supported by animal experiments. However, it should be noted that the human epidemiological evidence for the direct association between TFA exposure at various stages (perinatal and adolescent) and ADHD outcomes remains limited, requiring further investigation. Future studies should address the dose-response relationship of TFA exposure to ADHD to define safe TFA exposure limits. In clinics, researchers can develop better prevention and treatments by integrating evidence from genetic, environmental, and epigenetic studies, which will facilitate early prevention of ADHD and improve the prognosis of patients.
